# Chocolate Consumption and Risk of Heart Failure: A Meta-Analysis of Prospective Studies

**DOI:** 10.3390/nu9040402

**Published:** 2017-04-20

**Authors:** Fei Gong, Shuyuan Yao, Jing Wan, Xuedong Gan

**Affiliations:** Department of Cardiology, Zhongnan Hospital of Wuhan University, Wuhan University, Wuhan 430071, China; gongfei2017@163.com (F.G.); syyao2016@163.com (S.Y.); wanjing2017@163.com (J.W.)

**Keywords:** chocolate consumption, heart failure, meta-analysis, prevention

## Abstract

Epidemiological studies have shown inconsistent findings on the association between chocolate consumption and risk of heart failure (HF). We, therefore, performed a meta-analysis of prospective studies to determine the role of chocolate intake in the prevention of HF. We searched databases of PubMed, Web of Science, and Scopus through December 2016 and scrutinized the reference lists of relevant literatures to identify eligible studies. Study-specific hazard ratios (HRs) and 95% confidence intervals (CIs) were aggregated using random effect models. The dose–response relationship between chocolate consumption and incident HF was also assessed. This meta-analysis is registered with PROSPERO, number CRD42017054230. Five prospective studies with 106,109 participants were finally included. Compared to no consumption of chocolate, the pooled HRs (95% CIs) of HF were 0.86 (0.82–0.91) for low-to-moderate consumption (<7 servings/week) and 0.94 (0.80–1.09) for high consumption (≥7 servings/week). In dose–response meta-analysis, we detected a curve linear relationship between chocolate consumption and risk of HF (*p* for nonlinearity = 0.005). Compared with non-consumption, the HRs (95% CIs) of HF across chocolate consumption levels were 0.92 (0.88–0.97), 0.86 (0.78–0.94), 0.93 (0.85–1.03), and 1.07 (0.92–1.23) for 1, 3, 7, and 10 servings/week, respectively. In conclusion, chocolate consumption in moderation may be associated with a decreased risk of HF.

## 1. Introduction

Heart failure (HF) is a complex clinical syndrome characterized by dyspnea, fatigue, and fluid retention. Globally, there is an estimated 20 million people suffering from this syndrome, with a projected increase of 25% in prevalence in 2030 [1]. Despite advances in management, HF remains a major cause of mortality and morbidity worldwide, imposing a huge burden on the health care system [2]. Therefore, improving primary prevention of HF is of great importance for public health.

Previous studies demonstrated that consumption of chocolate products may confer salutary cardiovascular effects [3]. It is found that both acute and chronic chocolate intake reduce blood pressure [4], which is a major risk factor for HF. In addition, chocolate consumption has been shown to be associated with lower incidence of myocardial infarction and stroke [5], lower cardiovascular mortality [6], and improved vascular function in HF patients [7]. However, there are limited studies focusing on the association between chocolate consumption and risk of HF, and their results remain inconsistent. We, therefore, carried out a dose–response meta-analysis to determine the role of chocolate intake in prevention of HF.

## 2. Materials and Methods

### 2.1. Search Strategy

This study was conducted in accordance with the Meta-analysis Of Observational Studies in Epidemiology guideline [8]. We performed a comprehensive search in databases of PubMed, Web of Science, and Scopus through December 2016 to identify eligible studies, using the search terms “chocolate” or “cocoa” in combination with “heart failure”, “cardiac failure”, or “cardiac dysfunction”. Moreover, the reference lists of relevant literatures were manually reviewed to find additional articles. The protocol for this meta-analysis is available in PROSPERO (registration code: CRD42017054230).

### 2.2. Criteria for Inclusion

Studies were included if they met all of the following conditions: (1) the study was cohort study, case-control study, or post hoc analysis of randomized trials; (2) the exposure was chocolate consumption; (3) the outcome of interest was incident HF (or HF hospitalization/death); (4) the risk measures of HF, such as hazard ratios (HRs), were reported. For dose–response meta-analysis, the risk estimates should be provided for ≥3 quantitative categories of chocolate consumption, and the number of cases and person-years or participants for each category were also available (or information were provided to calculate them). Reviews, comments, abstracts, and duplicates were excluded.

### 2.3. Data Collection and Quality Assessment

Two reviewers independently abstracted the study characteristics. The maximally-adjusted risk estimates were also recorded for pooled analyses. We used the Newcastle-Ottawa Scale (NOS) [9] to assess the methodological quality based on three major domains: selection of participants, adjustment of confounders, and ascertainment of outcomes. A study with a NOS score ≥7 was considered as of high quality. Any discordance in data extraction and quality assessment were handled by consulting with a third reviewer.

### 2.4. Statistical Method

The aggregated risk estimates of HF were presented as HRs with 95% confidence intervals (CIs). To enable the pooling of data, we standardized chocolate consumption across studies using a common measure, i.e., servings/week. In the study by Kwok et al. [10] that reported chocolate consumption in g/day, the majority of participants consumed chocolate bars, and the average portion size for this type of chocolate was 50 g. Thus, we converted the intake into servings/week assuming that one serving contains 50 g of chocolate. For each study, we assigned the mean or median consumption of chocolate for the category to each corresponding HR. When the mean or median consumption was unavailable, we assigned the midpoint of the upper and lower boundaries in each category as the average consumption. If the upper boundary for the highest category was not reported, we assumed the midpoint as being 1.5-times the lower boundary. When the lowest category was open-ended, we set its lower boundary to zero.

We defined the low-to-moderate chocolate consumption as a median intake of <7 servings/week and high consumption as a median intake of ≥7 servings/week. The study by Lewis et al. [11] only provided an odds ratio of HF for chocolate consumption of ≥1 versus <1 serving/week. We have e-mailed the authors for additional detailed information but received no response; thus, that study was excluded from the final analysis. All included studies have reported more than one HR for the low-to-moderate chocolate consumption categories. For each of the studies, we first combined the HRs across levels of chocolate intake within the study by using a random-effect meta-analysis to derive a single HR for low-to-moderate chocolate consumption, as described in the study by Larsson et al. [12]. The study-specific HRs were then pooled for low-to-moderate and high chocolate consumption, respectively. The heterogeneity across studies was examined by the Cochrane *Q* test with a significant level of *p* < 0.1 and quantified using the *I*^2^ statistic. A value of *I*^2^ > 50% indicated the presence of high heterogeneity. Subgroup analyses were performed according to sex, body mass index (BMI), prior history of myocardial infarction, and follow-up durations, with differences between subsets confirmed by the Altman and Bland test [13]. We also conducted a sensitivity analysis by omitting each study one at a time. Publication bias was detected by using the Egger’s test.

To evaluate the dose–response relationship between chocolate consumption and risk of HF, we conducted a two-stage random-effects dose–response meta-analysis. In the first stage, we estimated a restricted cubic spline model with three knots at the 10th, 50th, and 90th percentiles of chocolate consumption using generalized least-square regression, considering the correlation within each set of published HRs as described by Orsini et al. [14]. Then the study-specific HR was pooled using the restricted maximum likelihood method in a random-effects meta-analysis [15]. We calculated a *p*-value for nonlinearity by testing the null hypothesis that the coefficient of the second spline is equal to zero. 

All statistical analyses were realized using STATA 12.0 (StataCorp, College Station, TX, USA) and R 3.2.5 (The R Foundation for Statistical Computing, Vienna, Austria) softwares, and *p*-values < 0.05 were regarded as of significance.

## 3. Results

### 3.1. Literature Search

**Figure 1:**
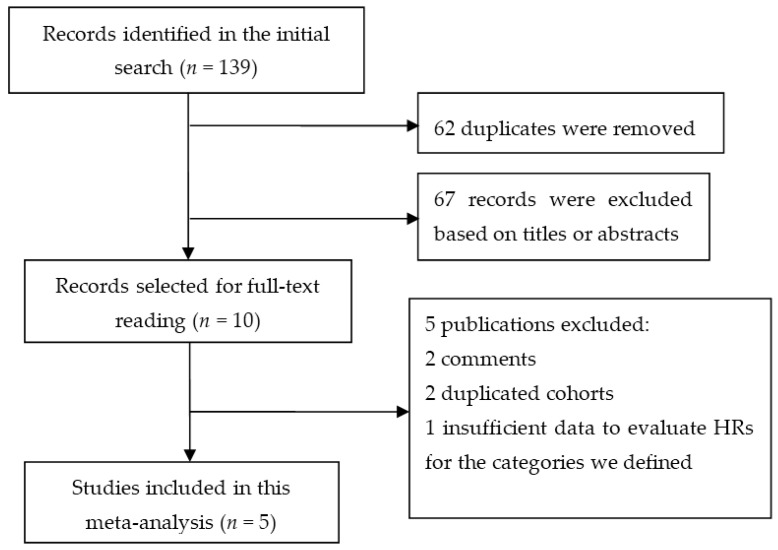
Flow diagram of study selection.

The search strategy initially identified 139 publications, of which 62 duplicates and 67 irrelevant studies were removed. Among the remaining 10 articles selected for full-text reading, 5 articles that failed to meet the eligibility criteria were excluded. Finally, five studies [10,16,17,18,19] that were published from 2009 to 2017 were included in this meta-analysis (Figure 1).

### 3.2. Study Characteristics

The baseline characteristics of the studies are displayed in Table 1. Briefly, the set of studies consists of four cohort studies and one post hoc analysis of a randomized trial, totaling 106,109 participants and 4832 HF cases. The mean age was 64 years, and men accounted for 59% of the total participants. Among the studies, the commonly used method for ascertainment of chocolate consumption was food frequency questionnaire, and HF events were frequently identified through ICD codes. Four studies were conducted in Europe, and the remaining one was performed in USA, with follow-up durations ranging from 9 to 14 years. The most commonly adjusted covariables among the included studies were age, BMI, total energy intake, smoking, and alcohol consumption. All studies had a high methodological quality, with a mean NOS score of 8.8.

### 3.3. Categorical Meta-Analysis

**Figure 2:**
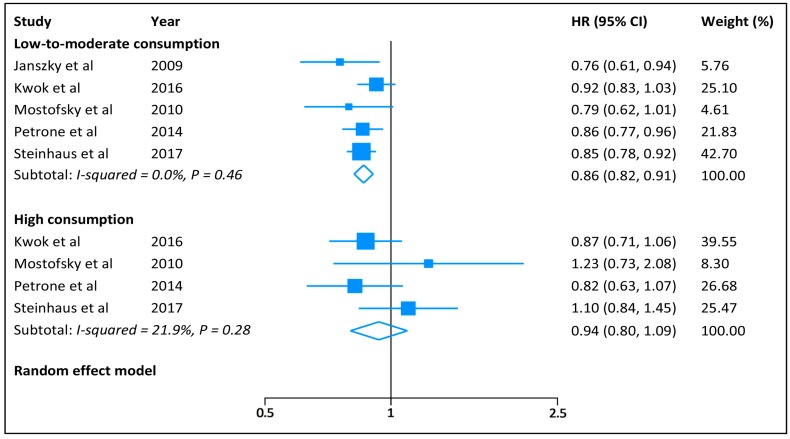
Hazard ratios of heart failure for low-to-moderate and high chocolate consumption.

**Figure 3:**
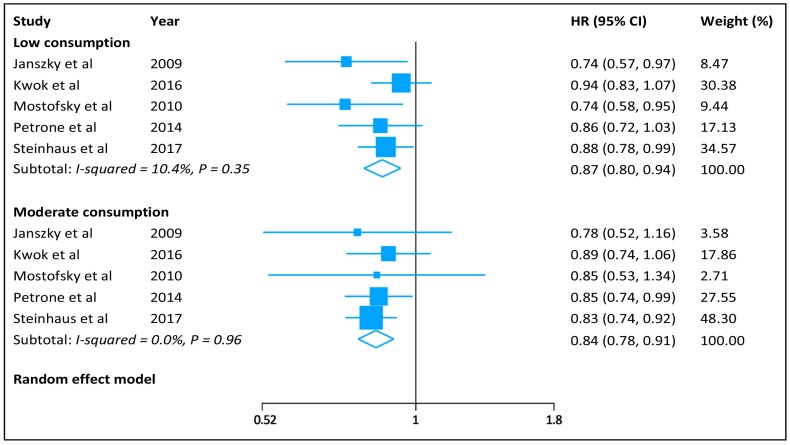
Hazard ratios of heart failure for low and moderate chocolate consumption.

The pooled HR (95% CI) of HF was 0.86 (0.82–0.91) for low-to-moderate chocolate consumption and 0.94 (0.80–1.09) for high consumption (Figure 2), with no significant heterogeneity across the studies (low-to-moderate consumption: *I*^2^ = 0.0%, *p* = 0.46; and high consumption: *I*^2^ = 22%, *p* = 0.28). When we separated low (<1 serving/week) from moderate (1–6 servings/week) chocolate consumption, the summarized HRs (95% CIs) were 0.87 (0.80–0.94) and 0.84 (0.78–0.91), respectively, for low and moderate consumption (Figure 3). In subgroup analyses, we observed no difference between subsets stratified by sex, BMI, prior history of myocardial infarction, and follow-up duration (Table 2). Sensitivity analysis by excluding individual study in sequence had no significant influence on risk of HF for low-to-moderate and high chocolate consumption. There was no evidence of publication bias (Egger’s test: *p* = 0.58).

### 3.4. Dose–response Meta-Analysis

**Figure 4:**
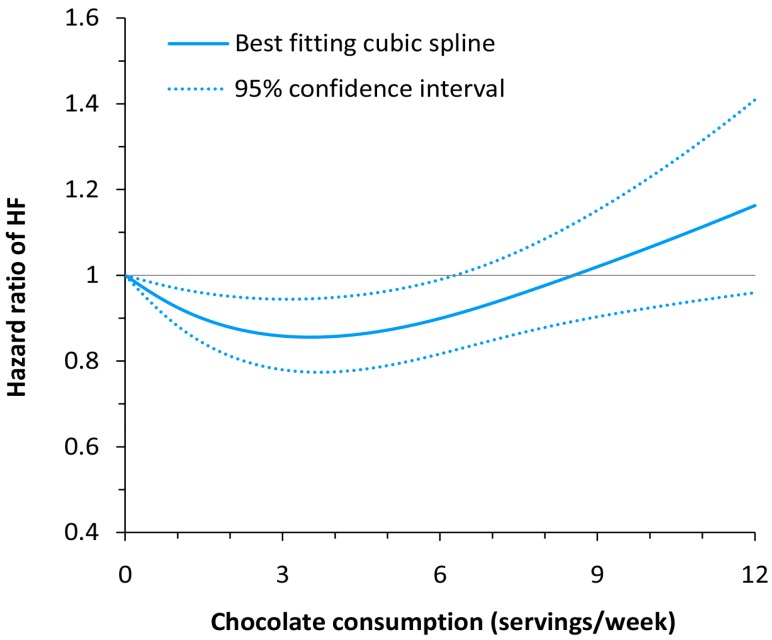
Dose–response relationship between chocolate consumption and risk of heart failure.

In dose–response meta-analysis, a curve linear relationship was found between chocolate consumption and risk of HF (*p* for nonlinearity = 0.005; Figure 4). Compared with non-consumption, the HRs (95% CIs) of HF across chocolate consumption levels were 0.92 (0.88–0.97) for 1 serving/week, 0.86 (0.78–0.94) for 3 servings/week, 0.93 (0.85–1.03) for 7 servings/week, and 1.07 (0.92–1.23) for 10 servings/week.

## 4. Discussion

There were limited studies that have investigated the association between chocolate intake and risk of HF. Our meta-analysis showed that light-to-moderate, but not high, consumption of chocolate was associated with a reduced risk of HF. Besides, we observed a nonlinear relationship between chocolate consumption and risk of HF in dose–response meta-analysis.

Chocolate is an important dietary source of flavonoids, a subclass of polyphenols. Previous studies have demonstrated that flavonoids in chocolate may be responsible for the salutary effects of chocolate on blood pressure, possibly by acting as an angiotensin converting enzyme inhibitor [20]. Flavonoids also offer improvements in other risk factors for HF, including increasing high-density lipoprotein cholesterol, reducing inflammation, and improving endothelial function [21,22]. In addition, it is well known that flavonoids have great antioxidant properties [23]. Experiments using human endothelial cells in culture have also indicated that flavonoids activates nitric oxide synthase, thus stimulating the generation of nitric oxide and contributing to the maintenance of normal cardiac function [24,25]. Therefore, these factors in combination may explain the protective role of chocolate against HF.

In dose–response meta-analysis, we found a J-shaped relationship between chocolate consumption and risk of HF. Initially, compared to no chocolate consumption, a moderate consumption is associated with a risk reduction in HF incidence of 16%. A higher than moderate intake is not associated with a decreased risk. This result may be attributable to the high calorie content of commercially available chocolate. When consuming chocolate in high amounts, the high-energy content of chocolate may lead to increased weight gain [26], a known risk factor for developing HF [27,28]. In addition, it is plausible that higher consumption of chocolate causes less energy intake from other foods that may be salutary for the prevention of HF. Thus, the benefits of flavonoids appear to be countered by the adverse effects of high energy intake when consuming excessive chocolate products. In three individual studies subgroups of BMI < 25 kg/m^2^ [18,19] and those with lower energy intake [10], a more linear pattern of risk reduction with more chocolate consumption was observed. However, in the overall results, all reported individual studies showed a J-shaped curve.

Our finding is in contrast to a recent meta-analysis [10], which showed that chocolate consumption was not associated with incident HF (HR: 0.87, 95% CI: 0.71–1.06). In that study, the authors only pooled the risk estimates of HF for the highest versus lowest category of chocolate intake, and because of considerable heterogeneity, were very prudent with results, pointing towards a non-significant benefit of consuming chocolate. A previous study [18] indicated that the association between chocolate consumption and HF was stronger in lean than in overweight/obese subjects. In the present study, we found no difference in risk of HF between subgroups with normal or elevated BMI. The reason for the lack of difference is unclear. However, this result should be treated with caution, given the limited studies available for subgroup analysis. Future studies concerning this issue are required.

Of course, some remaining points need to be addressed. In all of the included studies, the incidence of HF was reported as cumulative incidence in years, and we do not know the HF-free survival curves. Although our results suggest that chocolate consumption may play a role in the prevention against HF, we cannot derive from the data how many years of chocolate consumption are needed to reach this result, and cannot exclude the possibility that it may contribute only to delaying the occurrence of HF, even possibly even only by delaying the occurrence of other cardiac events that would trigger the development of HF. In addition, it is unclear whether the competing risk of death may affect our final results. Competing risk of death is an important consideration in geriatric studies [29]; however, none of the original studies have addressed the effect of this competing risk on HF incidence. Due to a lack of access to individual participant data, performing additional competing risk analysis in our work is also infeasible.

There are several limitations in our work. First of all, the recall and selection bias cannot be eliminated because of the observational nature of original studies. However, all included studies had a prospective design, and we only selected the maximally adjusted HRs for pooling analyses. Second, due to the lack of relevant data, we cannot evaluate the risk of HF for consuming different types of chocolate or different amounts of energy intake as was noted to have an interaction with the effects of chocolate consumption in the study of Kwok et al. [10]. For the latter interaction, we would argue that the HRs of the individual studies have been adjusted for energy intake, and are less likely to have influenced our results in this meta-analysis. Third, the lower HF incidence for low-to-mild chocolate consumption observed in our study may be associated with healthier dietary habits. Subjects consuming less chocolate may have healthier diets than those consuming higher amount of chocolate. Nevertheless, most dietary factors were not adjusted in the original studies. Fourth, all included studies were conducted in USA or Europe. Thus, generalization of our findings to other populations should be used with caution.

## 5. Conclusions

In summary, this meta-analysis indicates that chocolate consumption in moderation may be associated with a decreased risk of HF. Future studies are required to determine whether the association differs by chocolate subtypes or levels of energy intake and to clarify the mechanisms involved.

## Figures and Tables

**Table 1:** Baseline characteristics of the included studies.

Study	Design	Population	Participants (HF/total)	Ascertainments		Location	FU (Years)	Adjustments
				Exposure	Outcome			
Janszky 2009 [16]	Cohort study	Non-diabetic patients with post MI	279/1169	Self-reported consumption	ICD-9 and 10 codes	Sweden	8.7	Age, sex, smoking, drinking, obesity, physical activity, coffee consumption, educational attainment, and sweet score
Kwok 2016 [10]	Cohort study	General population	1101/20,922	Food frequency questionnaire	ICD-10 code	UK	12.5	Age, sex, education, BMI, social class, physical activity, smoking, drinking, dietary energy, MI, diabetes, arrhythmia, systolic blood pressure, cholesterol level, and heart rate
Mostofsky 2010 [17]	Cohort study	Women with no history of diabetes, HF, and MI	419/31,823	Food frequency questionnaire	ICD-9 and 10 codes	Sweden	9	Age, dietary energy, education, BMI, physical activity, smoking, drinking, living status, postmenopausal hormone use, family history of MI, and hypertension, and high cholesterol
Petrone 2014 [18]	Post hoc RCT	Male physicians in the Physician’s Health Study	876/20,278	Food frequency questionnaire	Self-reported diagnosis validated by medical records	USA	9.3	Age, BMI, smoking, drinking, exercise, dietary energy, and prevalent atrial fibrillation
Steinhaus 2017 [19]	Cohort study	Men with no history of diabetes, HF, and MI	2157/31,917	Food frequency questionnaire	ICD-9 and 10 codes	Sweden	14	Age, dietary energy, DASH diet component score, education, BMI, physical activity, smoking, drinking, family history of MI, hypertension, and high cholesterol

BMI, body mass index; DASH, Dietary Approaches to Stop Hypertension; FU, follow-up; HF, heart failure; ICD, International Classification of Disease; MI, myocardial infarction; RCT, randomized controlled trials.

**Table 2:** Subgroup analyses for the risk of heart failure.

Subgroup	Low-to-Moderate Chocolate Consumption				High Chocolate Consumption			
	No. of Reports	*I*^2^	HR (95% CI)	*p* for Interaction	No. of Reports	*I*^2^	HR (95% CI)	*p* for Interaction
Sex								
Men	3	0%	0.87 (0.82–0.92)	0.90	3	18%	0.93 (0.78–1.09)	0.88
Women	2	23%	0.88 (0.75–1.04)		2	35%	0.96 (0.66–1.39)	
BMI								
<25 Kg/m^2^	3	73%	0.91 (0.74–1.12)	0.84	3	54%	0.87 (0.59–1.28)	0.77
≥25 Kg/m^2^	3	0%	0.89 (0.82–0.96)		3	32%	0.93 (0.74–1.16)	
Prior MI								
No	4	11%	0.87 (0.82–0.93)	0.24	4	18%	0.94 (0.81–1.10)	0.55
Yes	2	0%	0.78 (0.66–0.93)		1	-	0.78 (0.43–1.42)	
Follow-up								
<10 years	3	0%	0.83 (0.76–0.91)	0.34	2	46%	0.94 (0.65–1.37)	0.92
≥10 years	2	23%	0.88 (0.81–0.95)		2	46%	0.96 (0.77–1.20)	

BMI, body mass index; CI, confidence interval; HR, hazard ratio; MI, myocardial infarction.
